# Factors affecting pathways to care for children and adolescents with complex vascular malformations: parental perspectives

**DOI:** 10.1186/s13023-022-02432-4

**Published:** 2022-07-15

**Authors:** Bryan A. Sisk, Anna Kerr, Katherine A. King

**Affiliations:** 1 Division of Hematology/Oncology, Department of Pediatrics, Washington University School of Medicine, 4523 Clayton Avenue, Campus Box 8005, St. Louis, MO 63110 USA; 2 Bioethics Research Center, Department of Medicine, Washington University School of Medicine, St. Louis, MO USA; 3 Department of Primary Care, Heritage College of Osteopathic Medicine, Ohio University, Athens, OH USA; 4 Division of Genetics and Genomic Medicine, Department of Pediatrics, Washington University School of Medicine, St. Louis, MO USA

**Keywords:** Pediatrics, Vascular malformation, Vascular anomaly, Health care access, Communication, Rare disease

## Abstract

**Background:**

Complex vascular malformations (VMs) are rare disorders that can cause pain, coagulopathy, disfigurement, asymmetric growth, and disability. Patients with complex VMs experience misdiagnosis, delayed diagnosis, delayed or inappropriate treatments, and worsened health. Given the potential consequences of delaying expert care, we must identify the factors that impede or facilitate this access to care.

**Results:**

We performed semi-structured interviews with 24 parents (21 mothers; 3 fathers; median age = 42.5 years) of children with complex VMs and overgrowth disorders living in the US, recruited through two patient advocacy groups – CLOVES Syndrome Community, and Klippel-Trenaunay Support Group. We performed thematic analysis to assess parental perspectives on barriers and facilitators to accessing expert care. We identified 11 factors, representing 6 overarching themes, affecting families’ ability to access and maintain effective care for their child: individual characteristics (clinician behaviors and characteristics, parent behaviors and characteristics), health care system (availability of specialist multidisciplinary teams, care coordination and logistics, insurance and financial issues, treatments and services), clinical characteristics (accuracy and timing of diagnosis, features of clinical presentation), social support networks, scientific progress, and luck and privilege. Additionally, access to information about VMs and VM care was a crosscutting theme affecting each of these factors. These factors influenced both the initial access to care and the ongoing maintenance of care for children with VMs.

**Conclusion:**

Parents of children with VMs report multiple factors that facilitate or impede their ability to provide their child with optimal care. These factors represent possible targets for future interventions to improve care delivery for families affected by VMs.

**Supplementary Information:**

The online version contains supplementary material available at 10.1186/s13023-022-02432-4.

## Introduction

Vascular malformations (VMs) are rare disorders that affect the development, structure, and/or function of vasculature in children [1, 2]. Some complex VMs are associated with syndromes caused by genetic variants that lead to overgrowth, such as Congenital Lipomatous Overgrowth, Vascular Malformations, Epidermal Nevis, Skeletal anomalies (CLOVES) and Klippel-Trenaunay syndromes [1, 3]. These complex VMs can cause pain, coagulopathy, disfigurement, asymmetric growth, and disability [3]. Furthermore, these disorders can lead to social stigmatization and myriad uncertainties for families [4–7].

In recent years, discoveries of genetic drivers for VMs have led to new disease classifications and novel treatment options. For example, somatic *PIK3CA* variants drive the growth of many VMs, as well as somatic overgrowth syndromes [8]. With these discoveries, many patients with VMs might now benefit from treatment with targeted inhibitors [9–11]. in addition to surgery and/or interventional radiology procedures.

Given this rapid evolution of knowledge and availability of novel treatments, it is imperative that patients with complex VMs receive expert medical care coordinated between primary and specialist teams. Pediatricians play a pivotal role in identifying vascular lesions, facilitating initial workup, providing referrals, and collaborating with specialists to maintain the child’s health. Due to the rarity of these conditions and complex presentation of symptoms, many pediatricians are unfamiliar with VMs, which can lead to misdiagnosis, delayed diagnosis, delayed or inappropriate treatments, and worsened health [12, 13]. Given the potential consequences of delaying coordinated care with VM specialists, it is essential to identify factors that impede or facilitate the ability of families to access and maintain this complex care. However, almost no studies have evaluated care delivery for patients with VMs, despite a growing body of research in other rare diseases [14–16]. In this qualitative study, we aimed to identify these barriers and facilitators from the perspectives of parents whose children have complex VMs.

## Results

### Participant characteristics

We performed 24 interviews, ranging from 39 to 73 min. Participating caregivers were predominantly White (n = 22, 92%), female (n = 21, 87), and had college or professional degrees (n = 8, 33% and n = 7, 29%, respectively). Parents’ ages ranged from 21 to 54 years (mean 41 years). (Table 1) Participants cared for children ranging in age from infancy to 16 years (median 11 years). (Table2) Approximately equal proportions of male and female children were represented, and all children’s care was covered by health insurance. Parents reported a high disease severity (median 8/10, Interquartile Range: 5–9) and interference of disease in the child’s life (median 7.5/10, Interquartile Range 6–9). Participants resided in 14 different states, representing multiple regions of the US.

**Table 1:** Participant demographics

Participant characteristic	n	(%)
Age in years	Median 42.5	Interquartile range 38–47
*Gender*		
Female	21	(87%)
Male	3	(13%)
*Race*^*a*^		
White	22	(92%)
Black or African American	1	(4%)
*Ethnicity*		
Hispanic, Latin, or Spanish Origin	4	(17%)
*Education*		
Some college	9	(38%)
College degree	8	(33%)
Graduate or professional degree	7	(29%)
*Household income*		
$24,999 or less	1	(4%)
$25,000–$49,999	3	(13%)
$50,000–$74,999	1	(4%)
$75,000–$99,999	6	(25%)
$100,000 or greater	11	(46%)
*Relationship status*		
Married or living as married	20	(84%)
Never married	2	(8%)
Divorced	2	(8%)

^a^Race and ethnicity responses were missing for one participant

**Table 2:** Child characteristics

Characteristic	n	(%)
Child’s age in years	Median 11	Interquartile Range 6–14
*Child’s gender*		
Boy	11	46
Girl	12	54
Child’s diagnosis		
*Lesion characteristics*^*a*^		
Lymphatic malformation	11	46
Venous malformation	10	42
Capillary malformation	8	33
Arteriovenous malformation	4	17
*Other physical manifestations*		
Hemangioma	3	13
Limb differences	1	4
Macrodactyly	1	4
*Associated syndromes or disorders*^*b*^		
CLOVES syndrome	9	38
Klippel-trenaunay syndrome	2	8
Fibro-adipose vascular anomaly	9	38
Kaposiform lymphangiomatosis	1	4
Macrocephaly-capillary malformation	1	4

Missing data from 1 participant for child characteristics.

^a^Not mutually exclusive.

^b^With the exception of Kaposiform Lymphangiomatosis, these disorders are often grouped in the larger diagnostic category of “PIK3CA-Related Overgrowth Spectrum.” However, many patients still identify their disorders by these historic terms

### Factors affecting access and maintenance of health care

We identified 11 factors, representing 6 overarching themes, affecting families’ ability to access and maintain effective care for their child. (Additional file 1: Fig. S1) Additionally, access to information about VMs and VM care was a crosscutting theme affecting each of these factors. See below and Table 3 for illustrative excerpts.

**Table 3:** Representative excerpts from transcripts

*Theme: Individual characteristics (Theme present in 24/24 interviews)*	
*Clinician behaviors and characteristics* Present in 24/24 interviews	“Having a pediatrician that is fully supportive of us getting the proper care, having a huge advocate from the neurologist locally from the get-go that took the time to do the research and learn about the syndrome to her full extent as possible” [CAR 44, father] “I always feel like I’m the one educating, including her pediatrician. She’s never seen anything like it before.” [CAR 20] “I hate to say it, but there’s a level of arrogance with some doctors. They don’t want to manage things from another doctor that says this. It’s hard.” [CAR 39] “I have found a huge reluctance in referring us to someone else. I feel like they want to keep us in-house and don’t want to refer us to someone else.” [CAR 33]
Parent behaviors and characteristics Present in 18/24 interviews	“I was like, “Something serious is going on within her leg, and this is what I need you to do. If you can’t do that, I need you to tell me now so I could go to a different hospital. Something is going on, and we need to get to the bottom of it.” [CAR 20] “The way that we handled this was really about my husband reading everything he could. He would do literature searches and tell me people to contact, and then I would reach out to them.” [CAR 48] “Although I may come across as a pain in the butt, I really am just trying to do my best to advocate for [child] and make sure that she has the care she needs.” [CAR 39]
*Theme: Health care system (Theme present in 24/24 interviews)*	
*Availability of specialist multidisciplinary teams* Present in 20/24 interviews	“The fact that no one, locally, knows much about it is a barrier. That we have to go across the country in order to get the care that we want with doctors who we feel like really, really know what this is.” [CAR 19] “Then if you’re trying to get a first-time appointment, it’s months out. Once you’re already an existing patient, if you need to be seen within a couple weeks, you’ll get that appointment. You see what I mean? It’s like first-time patient, they’re just overwhelmed with patients.” [CAR 20] “We have had lots and lots of problems with finding doctors in our area that are basically even really willing to work with her, because they don't understand the complexity of what she has wrong with her leg. Sometimes we've had cases where her medical care has required a local doctor to contact her specialist and some of them just don't really want to take the time to do that, I don't think, just because some of the things that she's had to deal with are really outside their [laughter] box and capabilities.” [CAR 24]
*Care coordination and logistics* Present in 17/24 interviews	“It’s been difficult. The administrative part has been I think the most difficult part to try to get things done and make sure that everything was pre-approved before we got there. Then sometimes, we’d get there, and when we weren’t pre-approved, it was just sometimes it’s been messy. I guess that’s pretty stressful when we’re already doing it trying to go through these appointments with her.” [CAR 9] “Just not knowing how to navigate these systems, or who to call, or how you talk to a doctor, or how to pass information on. Just trying to understand how it was, just how the whole system worked, and then trying to manage others’ expectations was overwhelming.” [CAR 28] “It's just coordinating everything. It's hard and exhausting to try to explain to another doctor what another doctor said.” [CAR 31]
*Insurance and financial issues* Present in 13/24 interviews	“Every time we went to [city] everything was rejected. We paid for everything out of pocket for the first three years going to [city].” [CAR 38] “The compression garments, the massages. They say that’s all pay out of pocket,’cause insurance don’t cover that.” [CAR 11] “It’s just a huge worry. Because if you don’t have a good insurance, you’re not getting into these teams. You’re not. Then what? She’s seeing doctors that don’t know anything about it.” [CAR 20]
*Treatments and services* Present in 13/24 interviews	“It takes us about four hours to four and a half hours [drive] for a 30 min MRI. Then we travel back, or we’ll stay around the [city 2] area for a little bit. We do that because if we have the images taken in our state in [state 1], [state 2] can’t read them. The position of the slides that they take there, their interventional radiologists can read them. The ones from here, they cannot. It’s completely useless to even try to stay near.” [CAR 20] “The [imaging] equipment there is so much vastly different than what we have here. It’s all cutting edge, state of the art stuff… I know the equipment there is just much better, so I don’t mind [traveling there].” [CAR 38]
*Theme: Clinical characteristics (Theme present in 23/24 interviews)*	
*Accuracy and timing of diagnosis* Present in 18/24 interviews	“[Knowing the diagnosis] helped me in that I was able to concentrate our efforts into okay, so there’s a group of children who have this or people who have this. These are the things I need to look for health wise. These are the specialists I need to find. It gave me a lotta direction in that okay, so these are the things that could possibly happen. These are the preventative things that we need to be doing in regards to scanning and things of that nature. I was able to get the guidelines as to how to—not that how it’s treated because there’s no real treatment per se.” [CAR 1] “When we finally got the right diagnosis, it was a relief. It was bad news, but that was the best news to hear because we had an answer… It was relief, and then we have to now move forward with something known. It's so much better to know than to doubt everything that we were being told.” [CAR 31] “Then we went to a dermatologist, 'cause that's where you go for [other disease]. He said, "I don't know what this is. I can't help you, but I promise you it's not [that]." He sent us on our way.” [CAR 19]
*Characteristics of clinical presentation* Present in 18/24 interviews	“In our 20-week ultrasound the doctor found a mass. He didn't really know what it was at that point, and it was a little bit scary.” [CAR 43] “At birth, we could see that she had facial birthmarks. We thought it was bruising on her face from delivery, which didn’t go away” [CAR 10] “It just started to grow very rapidly. Then it became debilitating very rapidly once we found it. It was like wildfire.” [CAR 20]
*Theme: Social support networks (Theme present in 18/24 interviews)*	
*Social support networks* Present in 18/24 interviews	“Probably the biggest thing was having a community with people who you can talk to and hey, this is what I dealt with and what did you all experience? Or did you all go through the same thing?” [CAR 37] “I think it’s the first-person support and comradery in knowing you’re not alone… If they’ve not had the exact similar situation, they’ve probably had a very similar situation that could help guide you on what works for them or at least think the right questions to ask.” [CAR 34] “There are definitely things about the groups that have made me uncomfortable. There’s some, ‘You have to go see this one certain doctor because he’ll do things that are very outside of the norm, and he’ll be willing to do things that other doctors aren’t willing to do.’ I always take a step back from those sorts of situations.” [CAR 10]
*Theme: Scientific progress (Theme present in 12/24 interviews)*	
*Scientific progress* Present in 18/24 interviews	“I just don't ever feel like we're gonna make real progress here because there's just not enough money behind it, and there's not enough motivation to fix this… There's something here that does feel very hopeless at times. When you have a condition that's not fatal, the motivation [for research] doesn't feel there.” [CAR 36]” “There's this responsibility when you are—when you have an ultra-rare disease to be the guinea pig, and there's a benefit to us for being the guinea pig. You want to be the guinea, but sometimes you don't wanna be the guinea pig.” [CAR 36] “We found out that there’s a trial drug that was making a dramatic impact on [disease] patients… It took us about a year, maybe a year and a half, to get him on this trial drug, and he’s been on it for a year now. The drug has made a huge impact and positive impact on his life.” [CAR 44]
*Theme: Luck and privilege (Theme present in 8/24 interviews)*	
*Luck and Privilege* Present in 8/24 interviews	“I got in to see them because I had one of my physician friends call and share how debilitating my daughter was. Once we did that, we got in fairly quickly… [Without that,] it would have been terrible. It just would have been terrible.” [CAR 20] “I recognize that we are really, really fortunate that we have the ability to go across the country and do this… We have the ability to do that, but not everybody does.” [CAR 19] “I don’t know how parents do it who don’t have the education or the background to actually do the things that I have to do. If he was born to someone who wasn’t, I don’t know what they would have done living here in town where I’m living with the providers that we have. He probably wouldn’t have a diagnosis.” [CAR 1]
*Cross-cutting theme: information (Theme present in 16/24 interviews)*	
*Information* Present in 16/24 interviews	“I think it was pretty frustrating, hurtful, a lot of confusion. It was a whole, I guess, a time period of me just feeling like you can't get answers from doctors, you can't find anything online. It just like constantly looking for something that you can't get an answer to. It was quite a bit in the beginning I'd say. It was rough. It was rough.” [CAR 37] “Things getting paywalled some of the—and that was so just—it was so frustrating’cause you couldn’t share the information. I couldn’t share that with anyone, and having to pay for the studies is just really frustrating.” [CAR1] “It was really difficult. There's not a lot out there. Which is understandable because it's so rare. It wasn't very good time for me to be researching everything and then reading what's life expectancy and stuff like that, the negative parts about it.” [CAR 42] “I just look at everything that I can find, but again, even then I'm afraid to, I would really rather have that information from a doctor because there’s always that level of misinterpretation on my part. Not truly understanding the implications of some of the test results from the diagnosis and all that. I mean, I would rather get that information from a doctor, but nobody really has seemed to have that at all.” [CAR 33]

#### Individual characteristics

All parents (n = 24/24) described individual characteristics and behaviors of clinicians and parents. Clinician characteristics included knowledge, investment of effort, and helpfulness. Clinician behaviors such as facilitating referrals, escalating care, and providing validation/support were also important. Parents described how most non-specialist physicians lacked knowledge about their child’s disease. Parents found it helpful when physicians admitted their limitations and referred them to other specialists. Yet, some physicians were not willing to offer referrals or second opinions. Parents appreciated clinicians who advocated for their child and demonstrated commitment and investment. Some parents, however, did not find physicians who seemed dedicated to taking extra steps to help their family: “That’s one thing that I wish I had more, someone to lead the way. I felt lost. I felt alone. I didn’t know what to do. I felt completely helpless.” [CAR 20].

When clinicians were uninformed or dismissive, parents were burdened with doing their own research to educate clinicians and coordinate care. Consequently, parents’ own individual characteristics and behaviors supported their child’s care. Parents described a ceaseless drive to advocate for their child: “Out of desperation, I just felt relentless that I had to keep advocating and finding different avenues whenever we did find providers who wouldn’t help us or who didn’t know what to do.” [CAR 1] Several parents described the need to advocate because many clinicians lacked sufficient knowledge about these rare disorders. Advocacy included educating oneself about the diagnosis and speaking out on behalf of their child. This parental drive to advocate increased when clinicians lacked sufficient concern, treatments failed to improve symptoms, and physicians seemed to lack knowledge or competence.

#### Health care system

All parents (n = 24/24) described health care system factors, including access to multidisciplinary teams, care coordination, navigating insurance, and finding treatments and services locally. Given the rarity and complexity of these diseases, parents described the importance of multidisciplinary teams with expertise in VMs. Accessing these teams facilitated accurate diagnosis and treatment after parents struggled to find answers for months or years. These teams were critical because many physicians lacked knowledge of VMs, especially local, non-specialist physicians. However, most parents described difficulties accessing multidisciplinary teams. Many families had to travel long distances: “It’s hard. It’s 1000 miles one way.”[CAR 39] One parent was grateful they “only” had to drive 6 h to see their clinical team. For some, this distance prevented them from establishing continuous care. Also, parents often had to wait several months for an appointment with these specialist clinics.

Given the complexity of care and scarcity of experts, many parents described the burden of coordinating care. One parent called it a “full-time job.” [CAR 19] Another parent resigned from her professional job to coordinate her child’s care. To access multidisciplinary teams, parents needed to complete multiple forms and collect medical information from multiple sources. This process was intimidating for parents who lacked experience with the medical system. Parents also found it exhausting to coordinate information among physicians when their child’s care included multiple clinicians in different locations.

Seeking care from multiple doctors across different health care systems and states created challenges with insurance coverage and financial strains. Parents must pay multiple co-pays for visits at multidisciplinary clinics. Some parents were forced to transfer care to local clinicians with limited expertise in VMs, or pay out-of-pocket for care, due to insurance denials. Other parents had to contact the clinical team and insurance company repeatedly to ensure they received prior authorization for care. Furthermore, insurance often failed to cover essential equipment, such as compression garments or specialty shoes. Even with high-quality insurance coverage, many families still felt financial strain: “We're really fortunate to have insurance, and it's still been a financial burden. I can't imagine what it would be like if we didn't.” [CAR 29].

Parents often needed to seek treatments and services locally, at the direction of their specialist team. However, several parents described the challenges of accessing these services locally, especially in rural settings. Because of these limitations, some parents opted to travel long distances rather than rely on local services.

#### Clinical characteristics

Most parents (n = 23/24) described clinical characteristics, including achieving an accurate diagnosis and unique features of their child’s clinical presentation.

For many parents, an early and accurate diagnosis facilitated finding information about their child’s condition and identifying expert physicians. However, many families went months or years with inaccurate or incomplete diagnoses, leading to feelings of frustration and worry: “It's hard to describe how helpless you feel when you don’t know what it is. Because you don’t have a path forward.” [CAR 19].

Parents also described clinical features of their child’s condition that affected their pathway to care. Manifestations visible on the skin or observed on prenatal screening ultrasound often led to early evaluation, although early investigation did not necessarily lead to earlier diagnosis. In fact, it often resulted in numerous unnecessary tests and procedures. Additionally, severe and rapidly progressing symptoms led to early evaluation by clinicians.

#### Social support networks

Many parents (n = 18/24) described the role of social support networks, including social media and patient advocacy websites. Members of these social networks provided families with advice about clinicians, treatments, and symptoms. These groups provided reassurance to families as they navigated uncertainties. Some parents also received financial support from advocacy groups. Yet, parents also recognized the downsides of social networks, such as incorrect guidance and emotional distress from learning about other children with severe disease manifestations.

#### Scientific progress

Many parents (n = 12/24) described the state of scientific progress. Parents often reported the lack of existing information about their child’s condition, which limited their treatment options and their ability to understand how their child’s condition would progress. Some parents lamented a lack of interest from scientists and companies in researching these rare diseases. When research studies and clinical trials were available, some parents described how these studies provided pathways to achieving a diagnosis or receiving treatment. The paucity of research opportunities led some parents to feel obligated to participate in studies, which they perceived as both beneficial and burdensome.

#### Luck and privilege

Several parents (n = 8/24) described personal privilege and luck. Parents acknowledged how financial security permitted them to travel to distant medical centers and take time off work. Other parents benefitted from prior medical training. Even with these opportunities and privileges, several parents still attributed their ability to access care to luck. For example, one parent described encountering a physician who happened to read an article on the child’s disease the week before their appointment: “When you think about this journey, him having read that paper, if he hadn't, I don't know where we would be right now.” [CAR 36] Other parents described how their physicians had experience caring for one previous patient with this disease, or how they serendipitously met a clinician with interest and experience in VMs. Furthermore, some parents expressed gratitude that they happened to live near a multidisciplinary team.

#### The central role of information

Information played a central role in facilitating access and maintenance of expert care, influencing factors across all levels. Parents needed accurate information about the diagnosis, symptoms, progression, treatments, research opportunities, and experts in VMs to identify appropriate next steps. Yet, parents often received incomplete, inaccurate, or conflicting information from their clinicians. As a result, many parents relied on the internet and social media as information sources. Even when parents did locate information about VMs, they reported it was often difficult to find information relevant to their child’s unique medical needs.

Obtaining information was often the goal of parental advocacy, and information deficiencies reinforced the need for ongoing advocacy. Parents’ ability to find high-quality information was also affected by paywalls on scientific articles. Furthermore, the lack of scientific progress contributed to limited information: “I'll never forget, whenever I got that diagnosis, they gave me a printout that was two pages front and back. There was really, even on the internet, very, very limited information.” [CAR 37] Information about available studies and clinical trials (often from social media) also helped advance this science by recruiting families for studies. Without information, parents felt lost: “Just imagine being in a super dark hole and having no clue where to go.” [CAR 43].

## Discussion

**Fig. 1:**
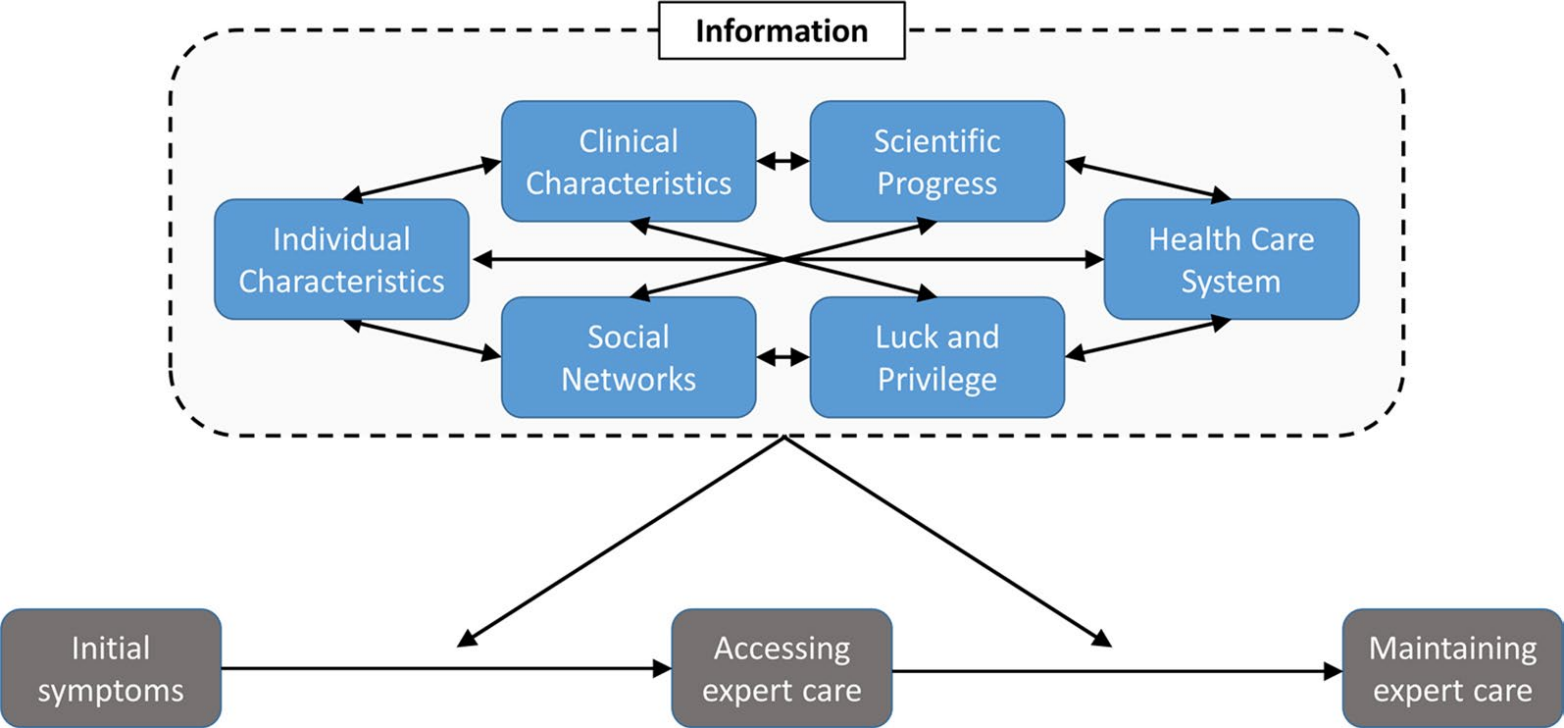
Factor affecting pathways to care

Parents of children with complex VMs identified barriers and facilitators to care that manifested across 6 levels ranging from individual behaviors to systemic policies and structures. (Fig. 1) Parental advocacy seems to play a disproportionately large role in families’ pathways to care, suggesting that parents of children with complex VMs might be at increased risk of caregiver burden and children with complex VMs might struggle to receive adequate care. Increased parental advocacy was a response to multilevel barriers, including lack of knowledge among clinicians, distance to multidisciplinary clinics, scarcity of local treatments and services, complex care coordination demands, and financial strains related to insurance coverage and travel costs. Health insurance coverage, in particular, created many challenges for families. For example, insurance policies dictated whether families could be seen by clinicians with VM expertise and how much this care would cost out-of-pocket. Furthermore, families spent great effort to ensure they received prior authorization for care to avoid unexpected and costly bills.

These burdens were enduring, meaning that even if families were able to initially access expert care, these barriers often prevented them from maintaining continuity of care. Consequently, families with lower household income, more restrictive insurance, or who live far from specialist teams are likely at a higher risk of delayed diagnosis and insufficient care. For example, in other rare diseases, distance from a tertiary center was associated with misdiagnosis and delayed diagnosis [17, 18] Future work should identify which families are at the highest risk of disparate medical care to support equitable health care delivery. Improving this care might require proactive outreach from VM specialists to community physicians. Additionally, specialists might leverage remote visits and telehealth to better coordinate generalist and specialist care for these families.

Parents identified information needs as a significant thread influencing many of these factors. Insufficient information is a common barrier in the context of rare diseases [19]. These limitations in quantity and quality of information can lead parents to scavenge multiple information sources, including peer support, internet searches, and social media [20, 21]. Information barriers in rare diseases have also been linked to delays in diagnosis and misdiagnosis [18, 22] Similarly, studies have demonstrated deficiencies in clinician knowledge about rare diseases and called for trustworthy, accessible information sources for clinicians [23] Parents of children with rare disorders frequently report knowing more about the disease than their clinicians, which can lead to complications in the clinical relationship and diminished trust [24, 25] Our findings suggest that, in the context of complex VMs, parents' increased advocacy and information-seeking results in them knowing more about the disease and system of care. Consequently, the burden of education and care coordination of care falls on parents. Future work should develop accessible and reliable sources of information for families affected by complex VMs and clinicians. For example, researchers and funding agencies might prioritize open access publications with accompanying lay summaries. Additionally, clinicians and researchers might develop and disseminate reliable medical information to families in print and video, while ensuring to incorporate families in this development process. Academic institutions could incentivize this work by considering these materials to be academic contributions that support career advancement and promotions.

Several factors were instrumental in helping families access expert care. Clinician advocacy and support played a significant role. Patients with rare diseases often encounter clinicians who are dismissive and unwilling to investigate symptoms or treatments further [26]. In the context of complex VMs, parents were grateful for clinicians who validated their concerns, researched symptoms and treatment options, facilitated referrals to specialists, and showed a genuine willingness to help. Social media also played a central role in helping parents find expert clinicians, achieve a diagnosis, learn about new treatments and research opportunities, and prepare for future symptoms and health needs. Patients with rare diseases are more likely than other health information consumers to rely on social networks for information [27]. Often, patients and parents turn to social media when they feel dismissed by clinicians. Social media also helps parents and families connect with similarly affected families and identify new research opportunities [28–30]. Finally, in the context of complex VMs, luck and privilege often plays a role in locating and maintaining expert care. Again, this finding suggests that future work should evaluate for potential health disparities in the care of complex VMs related to privilege based on location, income, education, and health literacy.

Our results should be interpreted in light of limitations. We recruited participants from patient advocacy groups with social media footprints. This approach allowed us to recruit geographically diverse participants and participants who have not been able to access expert care. However, our sample was predominantly White, female, and well educated with high incomes. Also, these families had already accessed patient support groups and have already arrived at a diagnosis. As such, our results might underrepresent the barriers that impede diagnosis, as well as access and maintenance of expert care. Future studies should aim to recruit purposively from advocacy groups and specialty clinics to ensure geographic, racial, and socioeconomic diversity. Parents of children with more severe disease might also be more likely to participate in patient advocacy groups. Furthermore, parents might have been affected by recall bias or conformity bias. Due to our recruitment strategy, we do not have access to additional clinical information that could inform the interpretation of these results. Lastly, we did not evaluate the perspectives of patients themselves, who might experience unique challenges and barriers as they transition to self-management in young adulthood.

## Conclusion

Parental interviews provided evidence for 6 themes of factors that facilitate or impede access to expert care for complex VMs. These factors manifest across multiple levels, ranging from individual behaviors to systemic structures and policies. Due to limited support, parents must strongly advocate for effective, coordinated care for their child when experiencing multilevel barriers to care. Future studies should aim to intervene upon these multilevel barriers to ensure equitable access to care for all patients with complex VMs.

## Methods

We report this study following Consolidated Criteria for Reporting Qualitative Research guidelines [31] (Appendix 1).

### Participants and recruitment

We interviewed parents of children with VMs, recruiting from two patient support groups: Klippel-Trenaunay (K-T) Support Group and CLOVES syndrome community. Caregivers were eligible if they were (1) 18 years or older, (2) spoke English, (3) lived in the US, (4) had a child younger than 18 years with a VM. No participants had clinical relationships with investigators. To engage these communities, we led an informational webinar and posted recruitment flyers via these organizations’ websites and social media platforms. We purposively sampled for fathers and racial minorities, but recruitment was affected by the limited diversity of the organizations’ memberships. To ensure thematic saturation, we aimed to recruit at least 20 participants [32]. We obtained verbal informed consent, and the institutional review board at Washington University approved this study.

### Data collection

Interviews occurred between June and October 2021. Participants completed a brief demographic survey in which race and ethnicity were self-reported. This survey included two questions that addressed the severity of illness and interference with their child’s life, with higher scores indicating greater severity and greater interference: (1) “On a scale of 1–10, to what extent do you think that your child's health problem is **severe**?”; (2) On a scale of 1–10, to what extent do you think that your child's health problem **interferes in their life**?” We conducted semi-structured interviews via telephone or video-conferencing software. We developed and refined the interview guide based on our prior wor [4–7, 33] and continued engagement with 3 parent advocates whose children had VMs. (Appendix 2) This guide explored characteristics of the child’s disease, barriers and facilitators to accessing medical care, and communication experiences. Two authors conducted interviews: BAS is a pediatric oncology physician specializing in VMs; AK is a medical educator with a PhD in communication. Only one interviewer was present during each interview. Both authors had led multiple prior qualitative studies. Interviews were audio-recorded and professionally transcribed.

### Data analysis

We performed descriptive statistical analysis of the demographic survey using Microsoft Excel 2016, calculating proportion, median, and interquartile range.

We employed thematic analysi [34]. to identify factors that influenced parental access to and maintenance of expert care. We adopted the Agency for Healthcare Research and Quality’s four components of access to care: coverage, services, timeliness, and workforce [35]. BAS and AK read all transcripts to familiarize themselves, then descriptively coded 5 transcripts to formulate preliminary categories and themes. The authors assigned each code to a category, then collapsed these categories into representative themes. These categories and themes were then refined through iterative cycles of independent coding and consensus meetings. After reviewing 10 transcripts, we reached saturation for representative themes. Using this finalized codebook (Table 4), BAS and AK then coded all 24 transcripts by independently coding transcripts, reviewing the other’s application of codes, marking disagreements, and resolving disagreements through discussion. A third coder (KAK) reviewed 25% of transcripts to assess for agreement with thematic coding and resolved any disagreements with BAS to confirm coding validity. We coded transcripts using Dedoose qualitative software. The results presented represent 100% coder agreement.

**Table 4:** Codebook definitions

Code	Definition
Clinician behaviors and characteristics	Descriptions of individual clinician (primary care and subspecialty) behaviors. Includes references how clinicians' knowledge, responsiveness, demeanor, collaboration, helpfulness, responsiveness, interest, and investment influence access to care, quality of care, and credibility
Parent behaviors and characteristics	Descriptions of parents as the driving force that leads to care. Includes references to persistence in learning/researching the condition, networking with others, educating clinicians, and advocating for the child. Also includes discussions of parental skill, attitude, intuition, and knowledge
Availability of specialist multidisciplinary teams	Descriptions of proximity to multidisciplinary specialist teams and availability of local care influence care. Includes references to travel distance, quantity/quality of local treatments and services, and rural health care
Care coordination and logistics	Descriptions of multidisciplinary care as a long process and/or one that is logistically complex. May refer to the burden of scheduling appointments, coordinating travel, requesting medical records, completing paperwork, and/or the lack of limited institutional support
Insurance and financial issues	Descriptions of financial strain related to travel, insurance, and treatments and services. Also, includes descriptions of financial privilege and the role of high-quality insurance in accessing and maintaining care
Treatments and services	Descriptions of availability of treatments and services. Include references to limited, ineffective, or harmful treatments, harsh side effects or morbidities, and uncertain outcomes. May include difficulty accessing supportive services or obtaining necessary equipment
Accuracy and timing of diagnosis	Descriptions of how receiving an early and accurate diagnosis affect care. May refer to diagnosis opening doors to research and support resources. Also includes references to the ways misdiagnosis or delayed diagnosis can lead to delayed care, inappropriate treatments, and emotional distress
Characteristics of clinical presentation	Descriptions of how the characteristics of the child’s disease can influence access to care. Includes references to severe symptoms or visible manifestations of the anomaly triggering earlier and more intensive diagnostic workups and late-onset or milder symptoms delaying care
Social support networks	Descriptions of the role of advice and recommendations from social support networks, including social media or other direct communication with families affected by similar diseases. Refers to the role social support plays in accessing care, often by identifying experts and treatments that are not widely publicized. Includes references to feeling reassured and supported
Scientific progress	Descriptions of how limitations of science and medical knowledge influence care. Include references to the lack of information in the medical community about newly discovered diseases. May also include descriptions of lack of dedication among researchers and pharmaceutical companies in expanding scientific knowledge
Luck or happenstance	Descriptions of the role of luck or chance in accessing care, often related to chance encounters with individuals who facilitated eventual diagnosis or access to experts. Includes chance encounters and privilege related to education, occupation, or geographical location

### Supplementary Information

**Additional file 1: Fig. S1**. Relationships between factors and themes

## Data Availability

The raw data generated and/or analyzed during the current study are not publicly available due ethical restrictions related to ensuring confidentiality, but de-identified data are available from the corresponding author on reasonable request.

## Appendix 1 COREQ Checklist and Additional Information

**Table:**

Domain 1: Research team and reflexivity		
*Personal characteristics*		
Interviewer/facilitator	Bryan Sisk (BAS) / Anna Kerr (AK)	Title page
Credentials	MD, MSCI / PhD	Title page
Occupation	Assistant Professor Pediatric Hematology and Oncology / Assistant Professor of Primary Care	Title page
Gender	Male/female	–
Experience and training	BAS and AK are trained in qualitative research methods and have published qualitative research with patient/family participants	Methods [6]
*Relationship with participants*		
Relationship established	BAS and AK held an informational webinar for interested participants	Methods [5]
Participant knowledge of the interviewer	BAS and AK introduced themselves and their roles in the project before each interview	–
Interviewer characteristics	Participant were informed that BAS is a pediatric oncology physician specializing in vascular anomalies and AK is a medical educator with a PhD in communication	Methods [5, 6]

Domain 2: Study design		
*Theoretical framework*		
Methodological orientation and theory	Thematic analysis	Methods [6]
*Participant selection*		
Sampling	Participants were recruited from two patient support groups	Methods [5]
Method of approach	Informational webinar and recruitment flyers via patient advocacy organizations’ websites and social media platforms	Methods [5]
Sample size	24	Results [7]
Non-participation	25 participants were contacted. 1 participant did not answer during the scheduled interview	–
*Setting*		
Setting of data collection	Data were collected via videoconference and phone interviews	Methods [5]
Presence of non-participants	Non-participants may have been present if participants completed the interview in their home or another public setting	–
Description of sample	3 men, 21 women. Ages ranging from 21 to 54 years. 22 identified as White, 1 as Black or African American. 4 were Hispanic, Latin, or Spanish. Data collected between June and October 2021	Results [7] & Table 2
*Data collection*		
Interview guide	Interviews were semi-structured. The guide explored the child’s disease, barriers and facilitators to care, and communication experiences	Methods [5]
Repeat interviews	None	–
Audio/visual recording	The interviews were audio-recorded	Methods (6)
Field notes	BAS and AK drafted memos after each interview of initial themes and insights	–
Duration	Interviews ranged in duration from 39 to 73 min (*M* = 60 min)	Methods [5]
Data saturation	We reached saturation after coding 10 interviews	Methods [5]
Transcripts returned	No	

Domain 3: Analysis an findings		
*Data analysis*		
Number of data coders	Three	Methods [6]
Description of the coding tree	Open coding, iterative thematic coding, and the coding validity check is described in methods	Methods [6]
Derivation of themes	We considered the Agency for Healthcare Research and Quality’s components of access to care while coding. However, final themes were derived during analysis	Methods [6]
Software	Dedoose	Methods [6]
Participant checking	None	–
*Reporting*		
Quotations presented	Participant quotations were used to illustrate the themes. All quotes were identified using the participant number	Results [7–12] & Table 3
Data and findings consistent	Data presented and the findings are consistent	–
Clarity of major themes	Major themes are presented clearly in text, table, and figure formats	Results [7–12], Fig. 1, & Table 3
Clarity of minor themes	Minor themes are presented in-text and in a table	Results [7–12] & Table 3

## Abbreviations

VM: Vascular malformation
CLOVES: Congenital lipomatous overgrowth, vascular malformations, epidermal nevis, skeletal anomalies
