# Supplementary figures and images for: Factors affecting pathways to care for children and adolescents with complex vascular malformations: parental perspectives
Source: Orphanet J Rare Dis. 2022 Jul 15;17:271. doi: 10.1186/s13023-022-02432-4 (PMC9287854; doi:10.1186/s13023-022-02432-4)

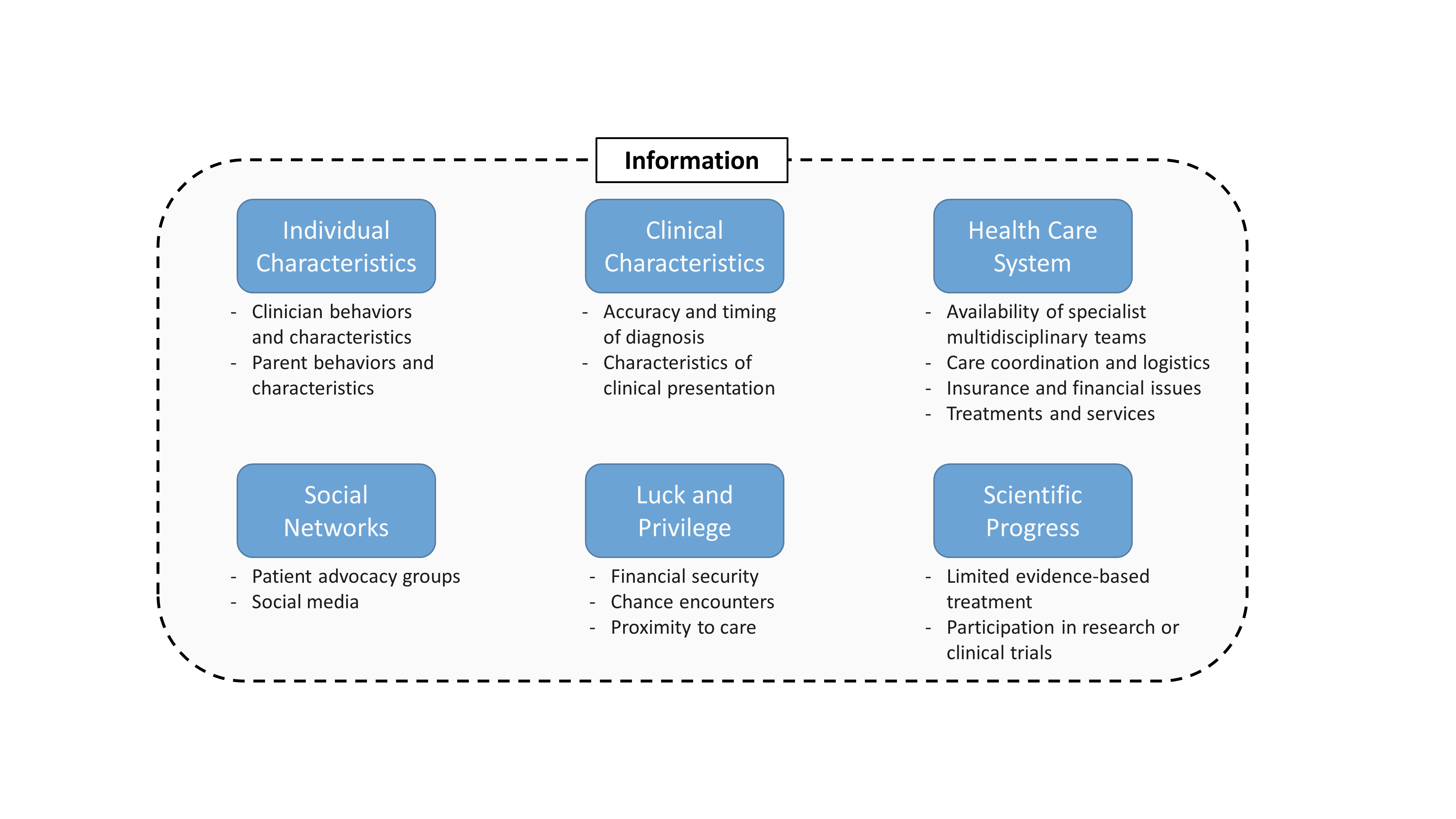

Supplement: Supplementary file 1 — Additional file 1: Fig. S1. Relationships between factors and themes [file 13023_2022_2432_MOESM1_ESM.tif]
